# SARS-CoV-2 S1 Protein Induces Endolysosome Dysfunction and Neuritic Dystrophy

**DOI:** 10.3389/fncel.2021.777738

**Published:** 2021-10-27

**Authors:** Gaurav Datta, Nicole M. Miller, Peter W. Halcrow, Nabab Khan, Timothy Colwell, Jonathan D. Geiger, Xuesong Chen

**Affiliations:** Department of Biomedical Sciences, University of North Dakota School of Medicine and Health Sciences, Grand Forks, ND, United States

**Keywords:** SARS-CoV-2 S1, endocytosis, neuron, neuritic dystrophy, endolysosomal acidification, lysosome

## Abstract

SARS-CoV-2 is the viral cause of the COVID-19 pandemic. Increasingly, significant neurological disorders have been associated with COVID-19. However, the pathogenesis of these neurological disorders remains unclear especially because only low or undetectable levels of SARS-CoV-2 have been reported in human brain specimens. Because SARS-CoV-2 S1 protein can be released from viral membranes, can cross the blood-brain barrier, and is present in brain cells including neurons, we tested the hypothesis that SARS-CoV-2 S1 protein can directly induce neuronal injury. Incubation of primary human cortical neurons with SARS-CoV-2 S1 protein resulted in accumulation of the S1 protein in endolysosomes as well as endolysosome de-acidification. Further, SARS-CoV-2 S1 protein induced aberrant endolysosome morphology and neuritic varicosities. Our findings suggest that SARS-CoV-2 S1 protein directly induces neuritic dystrophy, which could contribute to the high incidence of neurological disorders associated with COVID-19.

## Introduction

COVID-19 caused by SARS-CoV-2 infection continues to be associated with significant neurological disorders; 10–35% of COVID-19 patients manifest neurological symptoms that include anosmia, loss of taste, and cognitive difficulties (Garrigues et al., 2020; Hornuss et al., 2020; Liotta et al., 2020; Tenforde et al., 2020; Varatharaj et al., 2020). In severe cases of COVID-19 encephalopathy has been described (Helms et al., 2020a, b; Mao et al., 2020). COVID-19 symptoms can be long lasting (Sudre et al., 2021) and one-third of all COVID-19 patients manifest neurological and psychiatric symptoms 6-months following diagnosis of COVID-19 (Taquet et al., 2021). Even though SARS-CoV-2 can infect neurons directly (Song et al., 2021), the pathogenesis of the neurological disorders associated with SARS-CoV-2 infection remains unclear especially because only low or undetectable levels of SARS-CoV-2 virus have been reported in brain specimens (Matschke et al., 2020; Solomon et al., 2020; Lee et al., 2021).

Despite low levels of SARS-CoV-2 in brain specimens, SARS-CoV-2 viral proteins such as spiked glycoprotein (S protein) are present in brain tissues (Matschke et al., 2020; Meinhardt et al., 2021) and have been detected in brain neurons (Song et al., 2021). The S protein on the outer surface of SARS-CoV-2 is responsible for viral attachment to cells and for its internalization via cell surface receptors such as ACE2 (Yan et al., 2020) and neuropilin-1 (Cantuti-Castelvetri et al., 2020). S protein can be cleaved into S1 and S2 proteins by various cellular proteases, a process that is essential for viral entry into host cells (Hoffmann et al., 2020a, b; Ou et al., 2020; Shang et al., 2020). This cleavage results in S1 proteins disassociating from the virus and released S1 protein has been found in, for example, human urine (George et al., 2021). The concentration of shed SARS-CoV-2 S1 protein in bodily fluids and different tissues is difficult to determine, but it is predicted that the number of SARS-CoV-2 S1 protein molecules is higher than the number of virions because a single SARS-CoV-2 virion can shed about 300 monomeric spiked proteins (Bar-On et al., 2020). Exogenous S1 proteins can cross the blood-brain barrier and enter brain parenchyma (Rhea et al., 2021), S1 proteins contain the receptor binding domain (RBD) necessary for SARS-CoV-2 viral entry (Huang et al., 2020), and viral entry receptors such as ACE2 (Chen et al., 2020) and neuropilin-1 (Schwarz et al., 2008) are expressed in neurons, allowing S1 protein to be up taken into neurons via receptor-mediated endocytosis. Accordingly, we tested the hypothesis that S1 protein induces endolysosome and neuritic dysfunction in neurons.

## Materials and Methods

SARS-CoV-2 S1 proteins: The recombinant Receptor Binding Domain (RBD) of SARS CoV-2 S1 spike fusion protein (amino acids 319–541) with a N-terminal His tag and a C-terminal GFP tag (P2020-031) were obtained from Trenzyme GmbH, Konstanz, Germany and were used for localization studies. Tag-free S1 protein (aa Gln 14-Arg 685) was used for pH measurements and neuronal injury studies (Genscript, NJ, United States, Z03501-100). The recombinant full-length SARSCoV-2 S was obtained from R&D Systems (10549-CV). Tag-free S1 was heat-inactivated (95°C, 30 min) and was used as a control.

Cell cultures: Primary human cortical neurons were obtained from Neuromics (HNC001, Neuromics, Edina, MN) and were grown on T25 flasks coated with Neuromics Alpha BioCoat solution (AC001) in HNM0001 media according to manufacturer’s instructions. Neurons were grown for 5–7 days until confluent, split on Alpha BioCoat coated 24-well plates or glass bottom 35 mm^2^ dishes (MatTek), and were used for transfection and imaging studies. Neurons were provided by the manufacturer at passage 3 and used between passages 4–5 for all experiments. Mouse embryonic hippocampal E-18 cell line CLU199 (Cellutions Biosystems, Cedarlane, Ontario, Canada) were maintained at 37°C with 5% CO_2_ in 1x DMEM with 10% fetal bovine serum (FBS), 25 mM glucose and 1% penicillin/streptomycin according to manufacturer’s instructions. For our experiments, only cells from passages 3–7 were used. Primary mouse embryonic hippocampal neurons (E18) were obtained from E18 mouse cortex (C57EHP, BrainBits LLC, Springfield, IL) following standard primary neuron culture protocols and manufacturer’s instructions. Briefly, the tissue was digested with papain for 10 min and plated at 1,00,000 cells per well on either 12 mm poly-D-lysine coverslips (GG-12-PDL, Neuvitro Corporation, Vancouver, WA) in 24 well plates or on 35 mm^2^ ploy-D-lysine coated glass bottom dishes (P35GC-0-10-C, MatTek Life Sciences, Ashland, MA). NbActiv1 media (BrainBits) was used for both plating and maintenance, and neuronal cultures were incubated at 37°C with 5% CO_2_ with half-media exchanged every 3–4 days.

Plasmid transfections and transductions: The plasmid pCAG RpH-LAMP1-3XFLAG was a gift from Masssimilano Stagi (Addgene Plasmid # 163018; RRID: Addgene_163018)^1^. The bacterial culture was grown in LB media with ampicillin, and the plasmid isolated with Qiagen QIAprep Spin Miniprep Kit (27,104, Qiagen, MD, United States) was used for transfections. Neurons on 35 mm^2^ glass bottom dishes or 12 mm coverslips were transfected with 1μg plasmid DNA and 1 μL of Lipofectamine Stem transfection reagent for 48–72 h following which they were used for imaging and biological assays.

Live cell imaging of neurons: Neurons were transduced for 48–72 h with BacMam GFP transduction Control (B10383, Thermo Fisher Scientific for cytoplasmic GFP expression), BacMam Late Endosome RFP (for Rab7-RFP, C10589, Thermo Fisher Scientific), Lysosome-GFP/RFP (for LAMP1-GFP/RFP expression; C10507/C10504, Thermo Fisher Scientific), or stained for total endolysosomes with LysoTracker Red DND-99 (L7528, Thermo Fisher Scientific, 10 nM, 30 min at 37°C). The N-His-S1-RBD-GFP-C fusion protein was added to neuronal cultures (250 ng/mL) for 48 h following which the cells were imaged using a Zeiss LSM800 confocal microscope. Long term treatment with tag-free S1 protein was done for 72 h and neurons were then imaged by Zeiss LSM 800 confocal microscopy. All experiments were repeated at least three times independently, and a total of at least 30 neurons were analyzed. 3D reconstruction of images including lysosome size and distribution were performed with Imaris 9.6 software (Oxford Instruments, Bitplane, Zurich, Switzerland), and neuronal varicosities were analyzed from confocal images using NeuroLucida 360 software (MBF Bioscience, Williston, VT). Only those varicosities containing LAMP1 positive lysosomes and satisfying the measurement criteria (where maximum diameter of the varicosity is at least 2.5 times the diameter of the branch) were considered for calculations.

Endolysosome pH measurement: Lysosomal pH was measured in human cortical neurons transfected with the pCAG Rph-LAMP1-3XFLAG plasmid, which has the ratiometric pH sensitive indicator pHluorin fused to the pH non-sensitive mCherry. Neurons were transfected with 1μg plasmid DNA and 1 μL of Lipofectamine Stem transfection reagent for 48 h. Prior to imaging, the media was washed out and neurons were transferred to Hibernate E Low Fluorescence Medium (HELF, Brainbits) maintained at 37°C. The cells were imaged for one min (t-0) following which tag-free S1 protein was added and imaged at 1 min intervals for a total of 10 min. Heat-inactivated S1 protein was used as a control. The ratio of pHluorin (green) to mCherry (red) in regions of interest (ROI) was calculated using Image J. The ratio was converted to pH using an intracellular pH calibration kit (P35379, Thermo Fisher Scientific) with the addition of 10 μM nigericin and 20 μM monensin in Hibernate E Flow Fluorescence Media adjusted to different pH levels with HCl or NaOH.

Endolysosome pH in CLU199 cells was measured using a dextran-based ratio-metric method (Nash et al., 2019). CLU199 cells on 35 mm^2^ glass bottom dishes were loaded with10 μg/mL each of pH sensitive pHrodo Green Dextran (P35368, Thermo Fisher Scientific) and pH insensitive Texas Red Dextran (D1863, Thermo Fisher Scientific) for 24 h. Following washing with PBS, cells were transferred to Hibernate E Low Fluorescence Medium (HELF, Brainbits) and maintained at 37°C for imaging. Following treatments with either SARS-CoV-2 S1 protein or heat-inactivated SARS-CoV-2 S1 protein (boiled for 1 h), fluorescence emission was measured at 533 nm for Green dextran and 615 nm for Texas Red dextran. The ratio of 615/533 nm was converted to pH values using the same method as was described and used for human neurons. For all imaging and measurements of pH, we imaged a total of at least 5 fields under 40X magnification using a Zeiss LSM800 confocal microscope; at least 2–5 cells/field were imaged and three independent experiments were carried out.

Labeling of active endolysosomes: Active cathepsin D was labeled with BODIPY-FL Pepstatin A (P12271, Thermo Fisher Scientific). Cells were loaded with LysoTracker Red DND-99 (10 nM) along with BODIPY-FL Pepstatin A (1 μM) for 30 min in growth media maintained at 37°C, washed twice with PBS, and were imaged immediately after adding Hibernate E low fluorescence (HELF) media. Cells were imaged using a Zeiss LSM 800 confocal microscope and experiments were repeated three-times. Images were reconstructed as spots using Imaris 9.6 software (Oxford Instruments, Bitplane, Zurich, Switzerland), and object-based colocalization was carried at 0.2 μm distances. Active endolysosomes were calculated as the percentage of cathepsin D positive (BODIPY-FL Pepstatin A green) endolysosomes (LysoTracker Red) in relation to the total number of endolysosomes (LysoTracker Red).

Immunoblotting: CLU199 cells or primary mouse hippocampal neurons were plated on poly D-lysine coated 6-well plates at 1 × 10^6^/well or 0.5 × 10^6^/well, respectively. Cells were treated, harvested, and lysed in 1 × RIPA lysis buffer (Thermo Fisher Scientific) plus 10 mM NaF, 1 mM Na_3_VO_4_, and Protease Inhibitor Cocktail (Pierce). After centrifugation (13,000 × g for 10 min at 4°C), supernatants were collected, and protein concentrations were determined with a DC protein assay (Bio-Rad). Proteins (20 μg) were separated by SDS-PAGE (4–20% gel) and transferred to polyvinylidene difluoride membranes (Millipore). The membranes were incubated overnight at 4°C with rabbit anti-ACE2 (ab15348, Abcam, 1:500) followed by 2 TBS-T and TBS washes (5 min each). Appropriate rabbit secondary antibodies (LiCor) in LiCor blocking solution (TBS) were added for 1 h at room temperature. Blots were washed, and bands were visualized and analyzed using a LI-COR Odyssey Fc Imaging System.

Statistical Analysis: Data were shown as mean and SEM. All data were statistically analyzed with GraphPad Prism 9.0 software (GraphPad Software, Inc.). Statistical significance was determined by Student’s *t*-test or one-way ANOVA with Tukey *post hoc* tests. P value for statistical significance was set at 0.05.

## Results

**FIGURE 1:**
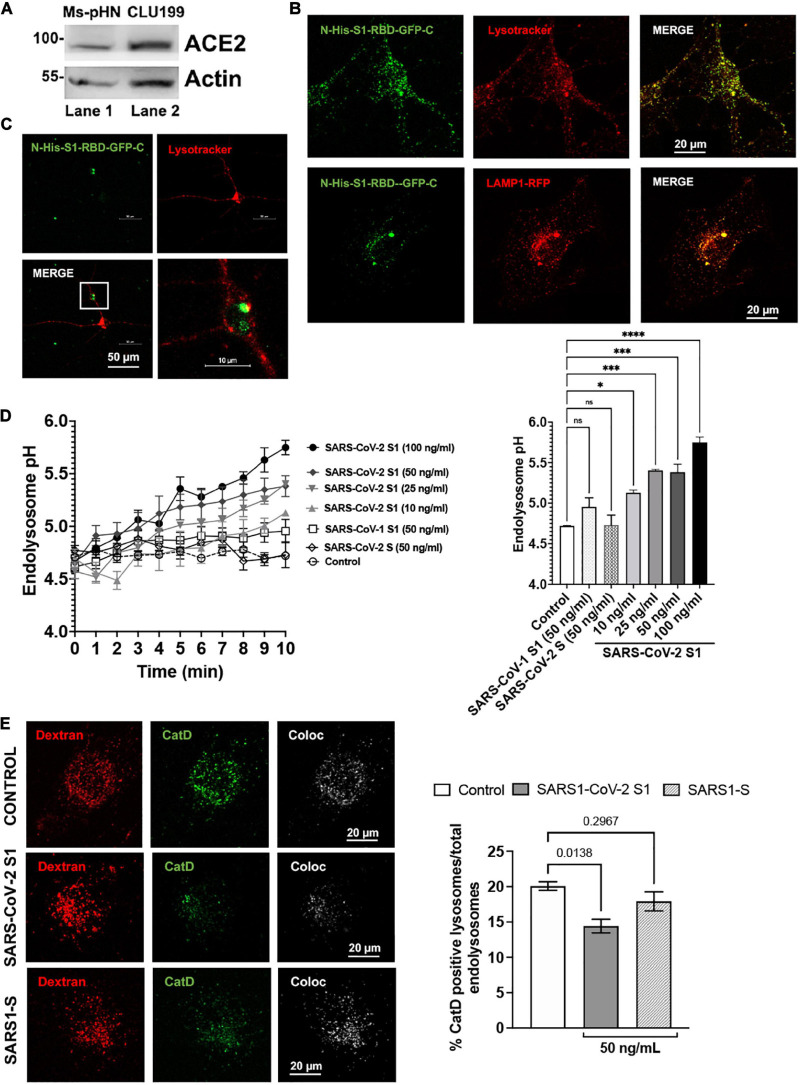
SARS-CoV2 S1 induced endolysosome dysfunction in mouse neuronal cells. **(A)** Immunoblotting showed the expression of ACE2 receptors (90 kDa) in mouse primary hippocampal neurons (Ms-pHN, Lane 1) and in mouse CLU199 hippocampal neuronal cells (Lane 2). **(B)** Representative confocal images show the presence of SARS-CoV2 S1 Receptor Binding domain (RBD) with C-terminal GFP (S1-RBD-GFP, 250 ng/mL for 30 min) in endolysosomes (LysoTracker Red) and lysosomes (LAMP1-RFP) lysosomes (red) in CLU199 cells. **(C)** Representative confocal images show the presence of S1-RBD-GFP (250 ng/mL for 30 min) in endolysosomes (LysoTracker Red) in primary cultured mouse hippocampal neurons. **(D)** Line graph shows that SARS-CoV2 S1 de-acidified endolysosomes in a concentration- and time-dependent manner. Whereas heat inactivated SARS-CoV2 S1 (control, 50 ng/ml), full length SARS-CoV2 S (50 ng/ml), or SARS-CoV1 S1 (50 ng/ml) did not induced endolysosome de-acidification. Bar graph shows that SARS-CoV2 S1 (compared with control at 10 min) de-acidified endolysosomes in a concentration-dependent manner (*n* = 3; *p < 0.05, ***p < 0.001, ****p < 0.0001). **(E)** Bar graph and representative confocal images show that SARS-CoV2-S1 (50 ng/ml for 30 min), but not SARS-CoV1 S1 (50 ng/ml), significantly decreased the percentage of active Cathepsin D positive lysosomes (BODIPY FL-Pepstatin A- green) vs. total endolysosomes (Dextran-Texas Red) (*n* = 3 repeats).

SARS-CoV-2 S1 induced endolysosome de-acidification in neurons. The effects of SARS-CoV-2 S1 protein on lysosome function was determined using primary neurons as well as neuronal cells of human and mouse origin. We demonstrated first that ACE2, a receptor for SARS-CoV-2 entry into host cells, was expressed on mouse primary hippocampal neurons and mouse CLU199 immortalized hippocampal neurons (Figure 1A). Given that the RBD necessary for SARS-CoV-2 viral entry into cells resides in the S1 protein (Huang et al., 2020), we next determined whether S1 protein could enter and reside in neuronal endolysosomes following receptor-mediated endocytosis. Incubation of CLU199 neurons for 30 min with a recombinant RBD of the S1 spike protein fused to a N-terminal His and a C-terminal GFP tag (S1-RBD-GFP) resulted in an accumulation of S1-RBD-GFP in LysoTracker-endolysosomes and LAMP1-positive lysosomes (Figure 1B). Similarly, primary cultured mouse hippocampal neurons incubated with S1-RBD-GFP (250 ng/ml for 30 min) resulted in an accumulation of S1 protein in LysoTracker-positive endolysosomes (Figure 1C). We next determined the extent to which accumulation of SARS-CoV-2 S1 protein accumulation affected endolysosome function. Because the acidic nature of endolysosomes is critical for optimum functioning of lysosomes, we first determined the effects of S1 proteins on endolysosome pH. The concentrations of S1 protein used were derived from our studies of concentration-dependent effects of S1 protein on endolysosome pH in CLU199 cells (Figure 1D). Heat-inactivated SARS-CoV-2 S1 (100 ng/ml) failed to de-acidify endolysosomes (Figure 1D) and in all subsequent studies heat-inactivated SARS-CoV-2 S1 was used as a control.

As a further measure of the specificity of the effects of SARS-CoV-2 S1 protein, we demonstrated that full-length SARS-CoV-2 S protein and the S1 protein (50 ng/ml) of SARS-CoV-1, which led to the SARS outbreak in 2002–2003, did not de-acidify endolysosomes (Figure 1D). To further assess lysosome function, we determined the percentage of lysosomes containing the pH-sensitive enzyme cathepsin D. In CLU199 neuronal cells (Figure 1E), SARS-CoV-2 S1 (100 ng/ml) treatment for 30 min, but not heat-inactivated SARS-CoV-2 S1 protein or SARS-CoV-1 S1 protein, decreased the percentage of endolysosomes containing enzymatically active cathepsin D.

**FIGURE 2:**
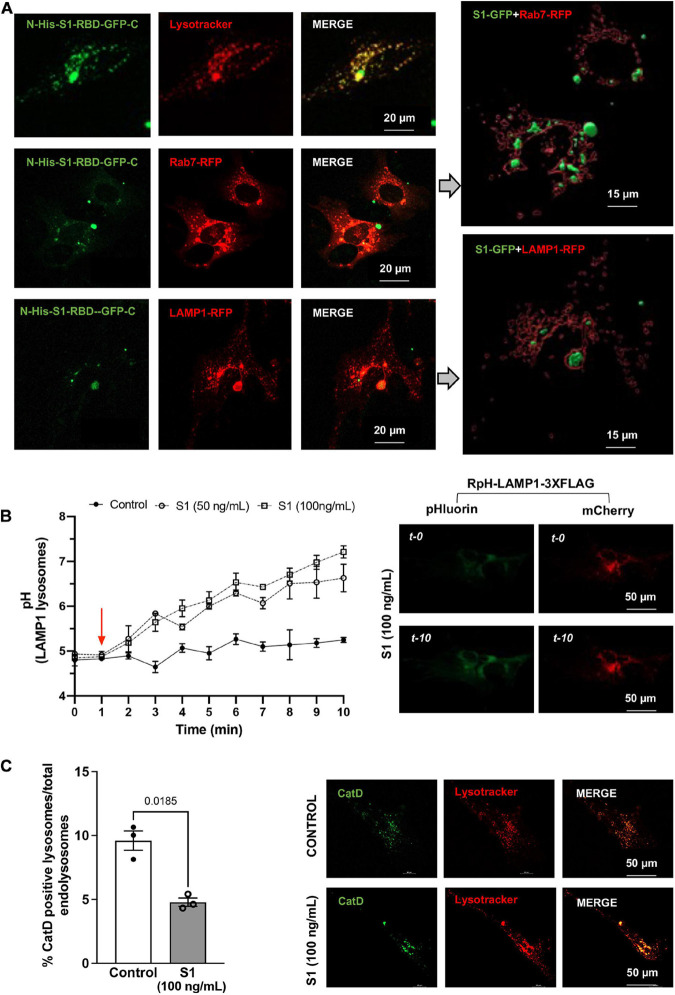
SARS-CoV2 S1 protein entered endolysosomes and induced lysosome dysfunction in human neurons. **(A)** Representative confocal images show the accumulation of SARS-CoV2 S1 Receptor Binding domain (RBD) with C-terminal GFP (S1-RBD-GFP, 250 ng/mL for 48h) in endolysosomes of primary human cortical neurons. Endolysosomes were identified with LysoTracker Red DND-99 or transduced with Rab7-RFP for late endosomes and Lysosome-RFP for lysosomes. 3D Imaris reconstruction of images show the presence of S1-RBD-GFP inside late endosome (Rab7-RFP) and lysosomes (LAMP1-RFP). **(B)** Graph shows time-dependent lysosome de-acidification effects of SARS-CoV2 S1 (50 and 100 ng/ml) in primary human neurons and lack of de-acidification effects of heat-inactivated S1 (controls). Lysosome pH was measured ratio-metrically by transfecting with RpH-LAMP1-3XFLAG. Representative confocal images of RpH-LAMP1-3XFLAG transfected human primary neurons at 0- and 10-min were shown on the right (*n* = 3 repeats). **(C)** Representative confocal images and bar graph show that SARS-CoV2 S1 (100 ng/ml for 30 min) decreased the percentage of active cathepsin D positive lysosomes (BODIPY FL-Pepstatin A-green) vs. total endolysosomes (LysoTracker Red) in primary human cortical neurons (*n* = 3).

Following characterization on the effect of SARS-CoV-2 S1 on endolysosome function in CLU199 neuronal cells, we determined further the extent to which SARS-CoV-2 S1 affect endolysosome function in primary human cortical neurons. We first confirmed the presence S1-RBD-GFP (250 ng/ml for 48 h) in Lysotracker-, Rab7-, and LAMP1-positive endolysosomes in primary human cortical neurons (Figure 2A). We next determined the extent to which accumulation of SARS-CoV-2 S1 protein accumulation affected endolysosome function in human neurons. Here, endolysosome pH was measured in neurons transfected with RpH-LAMP1-3XFLAG using a ratio-metric in LAMP1-positive vesicles as described (Ponsford et al., 2021). In human cortical neurons, S1 protein (50 and 100 ng/mL) de-acidified endolysosomes rapidly and robustly (Figure 2B). To further assess lysosome function, we determined the percentage of lysosomes containing the pH-sensitive enzyme cathepsin D. Similar to that observed in CLU199 neuronal cells, SARS-CoV-2 S1 (100 ng/ml) treatment for 30 min decreased the percentage of endolysosomes containing enzymatically active cathepsin D in human cortical neurons (Figure 2C).

**FIGURE 3:**
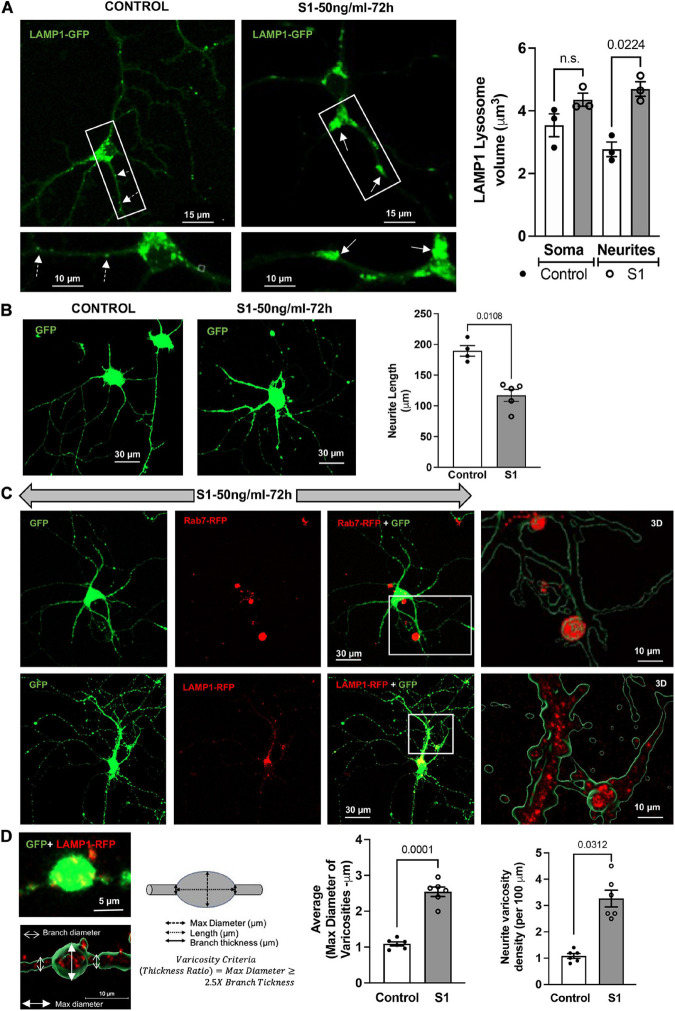
SARS-CoV2 S1 induced lysosome enlargement and neurite dystrophy. **(A)** SARS-CoV2 S1 (50 ng/ml for 72 h) induced morphological changes of lysosomes (LAMP1-GFP) in neuronal soma and along the neurites of mouse primary hippocampal neurons (DIV5-7) transduced with GFP; heat-inactivated S1 was used as a control. Bar graph shows that SARS-CoV2 S1 (50 ng/ml for 72 h) induced significant enlargement of lysosomes along the neurites but not in the soma (*n* = 3). **(B)** SARS-CoV2 S1 (50 ng/ml for 72 h) significantly reduced the length of neurites in primary mouse hippocampal neurons transduced with GFP; see representative confocal images and bar graph (*n* = 5). **(C)** Representative confocal images and 3D reconstruction (right panel) show the presence of late endosomes (Rab7-RFP) and lysosomes (LAMP1-RFP) in varicosities induced by S1 protein (50 ng/ml for 72 h) in primary mouse hippocampal neurons transduced with cytosolic GFP. **(D)** Varicosity-like structures along the neurites were observed in S1 treated neurons transfected with GFP and LAMP1-RFP. Bar graph shows that SARS-CoV2 S1 (50 ng/ml for 72 h) significantly increased the diameter and density of varicosities along the neurites of S1 treated neurons (*n* = 6).

SARS-CoV-2 S1 induced endolysosome enlargement and neurite dystrophy. We next determined longer-term effects of SARS-CoV-2 S1 protein on endolysosome structure and neurite morphology. Primary mouse hippocampal neurons that express ACE2 (Figure 1A) and accumulate S1-RBD-GFP in endolysosomes (Figure 1C) were transfected with LAMP1-GFP and then treated with S1 protein (50 ng/ml for 72 h); endolysosome volumes were significantly increased especially those along neurites (Figure 3A). Because neurons have extensive processes that require constant vesicular membrane trafficking to maintain axonal and somatodendritic plasma membrane domains, we determined the extent to which S1 proteins affect neurite morphology. In neurons expressing cytosolic GFP, S1 protein (50 ng/ml for 72 h) induced a significant reduction in neurite length (Figure 3B). Furthermore, in neurons co-expressing cytosolic GFP and lysosome markers (Rab7-RFP or LAMP1-RFP), S1 protein (50 ng/ml for 72 h) promoted the formation of varicosity-like structures filled with enlarged endolysosomes (Figure 3C). Finally, S1 protein (50 ng/ml for 72 h) significantly increased the average diameter and density of varicosities along the neurites (Figure 3D).

## Discussion

The main finding of our study was that exogenous SARS-CoV-2 S1 protein, which enters neurons via receptor-mediated endocytosis, induced endolysosome dysfunction and neurite dystrophy in neurons; such a finding provides evidence that SARS-CoV-2 S1 protein could directly induce neuronal injury.

SARS-CoV-2 S1 protein is derived from the spike glycoprotein (S protein) on the outer surface of SARS-CoV-2; this process that is catalyzed by various cellular proteases is essential for viral entry into host cells (Hoffmann et al., 2020a, b; Ou et al., 2020; Shang et al., 2020). Upon protease cleavage, S1 disassociates from viral membranes and is released (George et al., 2021), whereas S2 protein remains on the viral membrane. Thus, it is physiologically relevant to use recombinant SARS-CoV-2 S1 protein to study effects on the cell biology of target cells. On the other hand, both full-length SARS-CoV-2 S proteins and S2 proteins are membrane bound proteins, using recombinant full-length S proteins or S2 proteins may not be physiologically relevant in natural SARS-CoV-2 infection. However, recombinant full-length S proteins may be physiologically relevant in case of mRNA vaccine for COVID-19, because both Moderna’s mRNA-1273 and Pfizer/BioNTech’s BNT162b2 encode the full-length SARS-CoV-2 S protein (Corbett et al., 2020).

S1 protein contains the receptor binding domain (Huang et al., 2020) for receptors responsible for viral entry including ACE2 (Yan et al., 2020) and neuropilin-1 (Cantuti-Castelvetri et al., 2020). In addition, SARS-CoV-2 S1 protein can also bind to other receptors through specific and non-specific interactions (Hassanzadeh et al., 2020; Rhea et al., 2021). As such, SARS-CoV-2 S1 protein could interact with various types of cells in brain and/or enter those cells via endocytosis. In brain, SARS-CoV-2 virus and S1 protein have been shown to alter blood-brain barrier function (Buzhdygan et al., 2020; Reynolds and Mahajan, 2021). In addition, SARS-CoV-2 is neurotropic (Ramani et al., 2020; Song et al., 2021), and a recent study has identified the presence of S proteins in cortical neurons of COVID-19 patients (Song et al., 2021). The presence of S proteins in neurons could result from neuronal infection of SARS-CoV-2 or from internalization of shed S1 protein. Because both ACE2 (Chen et al., 2020) and neuropilin-1 (Schwarz et al., 2008) are expressed in neurons, both SARS-CoV-2 virus and shed S1 protein could directly induce neuronal injury. However, only low or undetectable levels of SARS-CoV-2 virus itself have been found in brain specimens (Matschke et al., 2020; Solomon et al., 2020; Lee et al., 2021). Thus, direct neuronal injury resulting from SARS-CoV-2 infection might be limited. On the contrary, a single SARS-CoV-2 virion can shed about 300 S1 proteins (Bar-On et al., 2020) and such shed S1 protein can cross the blood-brain barrier (Rhea et al., 2021). As such, we speculate that brain levels of S1 protein (derived locally or peripherally) are higher than the number of SARS-CoV-2 virions present in an infected patient, and that shed S1 proteins may play an important role in neuronal injury. Indeed, we demonstrated that exogenously added S1 protein induced neurite dystrophy. Our findings suggest S1 protein-induced endolysosome de-acidification and dysfunction may play an important role in the development of neurite dystrophy.

Endolysosomes form an integral part of the SARS-CoV-2 infection process, playing a role in its internalization into host cells (Burkard et al., 2014; Hoffmann et al., 2020a, b; Shang et al., 2020; Bayati et al., 2021) as well as in its egress from host cells (Ghosh et al., 2020). Other SARS-CoV-2 proteins, such as ORF7 and ORF3a, have been shown to disrupt endolysosome function (Hayn et al., 2021). A change in endolysosome pH, which is critical for optimum functioning of endolysosomes including degradative capability and mobility, potentially plays a crucial role in affecting neuronal function and the development of neurological symptoms, because neurons possess extensive processes that require constant vesicular membrane trafficking to establish and maintain axonal and somatodendritic plasma membrane domains. Being at the crossroads of transporting proteins to the synaptic terminals and in the degradation and recycling of dendritic cargos, endolysosomes have been shown to be important in modulating neurite morphology (Lee et al., 2011; Gowrishankar et al., 2015; Kulkarni and Maday, 2018; Farfel-Becker et al., 2019). Our finding that SARS-CoV-2 S1 induced endolysosome de-acidification and aberrant neuronal morphology in both human and mice primary neurons strongly suggest that SARS-CoV-2 S1 could induce neuronal injury directly, and support an earlier study showing mis-localization of the axonal protein Tau in infected neurons (Ramani et al., 2020).

S1 protein-induced neuronal varicosities could be resulting from endolysosome trafficking defects. Such S1 protein-induced endolysosome dysfunction could also disrupt the transport of synaptic vesicle precursors, lysosomes and autophagic components within both the axon and dendrites. Our results show that S1 protein enlarged lysosomes within these varicosities; a finding similar to that observed in Alzheimer’s disease (Gowrishankar et al., 2015; Gu, 2021). However, further mechanistic investigations are warranted to understand the exact nature of these structures and how they form. Because endolysosome trafficking in primary neurons is extremely complex and highly compartmentalized it is likely that endolysosome dysfunction, resulting from internalization of SARS-CoV-2 S1 proteins, leads to neuronal injury including alterations in neurite morphology and the formation of varicosities. Such direct neuronal injury effects of SARS-CoV-2 S1 could contribute, at least in part, to the high incidence of neurological and psychological manifestations in COVID-19 (Taquet et al., 2021).

In contrast, neurological symptoms associated with the 2003 SARS outbreak were only anecdotally reported (Chao et al., 2003; Ng Kee Kwong et al., 2020) and here we found that SARS-CoV-1 S1 protein, which enters neurons via same receptor-mediated endocytosis as does SARS-CoV-2 S1 protein, failed to de-acidify endolysosomes. Thus, the unique structure and sequence of SARS-CoV-2 S1 protein might induce endolysosome dysfunction and neuronal injury. It is not likely that ACE2-receptor binding domain of SARS-CoV-2 S1 mediates the endolysosome de-acidification effect in neurons, because the same ACE2-receptor binding domain is present in both SARS-CoV-2 S1 and SARS-CoV-1 S1 protein. It should be note that the current mRNA vaccine for COVID-19 (Moderna’s mRNA-1273 and Pfizer/BioNTech’s BNT162b2) encode the full-length S protein (Corbett et al., 2020). Based on our findings that full-length SARS-CoV-2 S protein did not elicit endolysosome de-acidification effect, no serious concern is raised for potential mRNA vaccine-induced neurological effect, which might depend on how much S1 proteins can be generated from mRNA encoded full-length S protein.

In summary, our findings suggest that SARS-CoV-2 S1 protein, which enters neurons via receptor-mediated endocytosis, induces endolysosome dysfunction and neurite dystrophy. Such findings provide evidence that SARS-CoV-2 S1 protein could directly induce neuronal injury and shed light on the high incidence of neurological disorders associated with COVID-19. However, the mechanisms whereby SARS-CoV-2 S1 protein induces endolysosome dysfunction remain unclear, and the extent to which and mechanisms by which SARS-CoV-2 S1 protein induces neuronal injury, directly or indirectly via glia cells, warrant further investigation.

## Data Availability Statement

The original contributions presented in the study are included in the article/supplementary material, further inquiries can be directed to the corresponding author/s.

## Ethics Statement

The animal study was reviewed and approved by the University of North Dakota Animal Care and Use Committee.

## Author Contributions

GD and XC designed the research. NM provided neuronal cell cultures. NK and PH preformed the initial experiments showing effects of S1 protein on endolysosome pH, TC conducted lysosome pH measurements. GD performed all experiments and analyzed the data. XC, GD, and JG wrote the manuscript. All authors contributed to the article and approved the submitted version.

## Conflict of Interest

The authors declare that the research was conducted in the absence of any commercial or financial relationships that could be construed as a potential conflict of interest.

## Publisher’s Note

All claims expressed in this article are solely those of the authors and do not necessarily represent those of their affiliated organizations, or those of the publisher, the editors and the reviewers. Any product that may be evaluated in this article, or claim that may be made by its manufacturer, is not guaranteed or endorsed by the publisher.

## Footnotes

1
http://n2t.net/addgene:16301
